# Electronic Media Use and Sleep Quality: Updated Systematic Review and Meta-Analysis

**DOI:** 10.2196/48356

**Published:** 2024-04-23

**Authors:** Xiaoning Han, Enze Zhou, Dong Liu

**Affiliations:** 1 School of Journalism and Communication Renmin University of China Beijing China

**Keywords:** electronic media, sleep quality, meta-analysis, media types, cultural difference

## Abstract

**Background:**

This paper explores the widely discussed relationship between electronic media use and sleep quality, indicating negative effects due to various factors. However, existing meta-analyses on the topic have some limitations.

**Objective:**

The study aims to analyze and compare the impacts of different digital media types, such as smartphones, online games, and social media, on sleep quality.

**Methods:**

Adhering to Preferred Reporting Items for Systematic Reviews and Meta-Analyses (PRISMA) guidelines, the study performed a systematic meta-analysis of literature across multiple databases, including Web of Science, MEDLINE, PsycINFO, PubMed, Science Direct, Scopus, and Google Scholar, from January 2018 to October 2023. Two trained coders coded the study characteristics independently. The effect sizes were calculated using the correlation coefficient as a standardized measure of the relationship between electronic media use and sleep quality across studies. The Comprehensive Meta-Analysis software (version 3.0) was used to perform the meta-analysis. Statistical methods such as funnel plots were used to assess the presence of asymmetry and a *p*-curve test to test the *p*-hacking problem, which can indicate publication bias.

**Results:**

Following a thorough screening process, the study involved 55 papers (56 items) with 41,716 participants from over 20 countries, classifying electronic media use into “general use” and “problematic use.” The meta-analysis revealed that electronic media use was significantly linked with decreased sleep quality and increased sleep problems with varying effect sizes across subgroups. A significant cultural difference was also observed in these effects. General use was associated with a significant decrease in sleep quality (*P*<.001). The pooled effect size was 0.28 (95% CI 0.21-0.35; *k*=20). Problematic use was associated with a significant increase in sleep problems (*P*≤.001). The pooled effect size was 0.33 (95% CI 0.28-0.38; *k*=36). The subgroup analysis indicated that the effect of general smartphone use and sleep problems was *r*=0.33 (95% CI 0.27-0.40), which was the highest among the general group. The effect of problematic internet use and sleep problems was *r*=0.51 (95% CI 0.43-0.59), which was the highest among the problematic groups. There were significant differences among these subgroups (general: *Q*_between_=14.46, *P*=.001; problematic: *Q*_between_=27.37, *P*<.001). The results of the meta-regression analysis using age, gender, and culture as moderators indicated that only cultural difference in the relationship between Eastern and Western culture was significant (*Q*_between_=6.69; *P*=.01). All funnel plots and *p*-curve analyses showed no evidence of publication and selection bias.

**Conclusions:**

Despite some variability, the study overall confirms the correlation between increased electronic media use and poorer sleep outcomes, which is notably more significant in Eastern cultures.

## Introduction

Sleep is vital to our health. Research has shown that high sleep quality can lead to improvements in a series of health outcomes, such as an improved immune system, better mood and mental health, enhanced physical performance, lower risk of chronic diseases, and a longer life span [1-5].

Electronic media refers to forms of media or communication that use electronic devices or technology to create, distribute, and display content. This can include various forms of digital media such as smartphones, tablets, instant messaging, phone calls, social media, online games, short video platforms, etc. Electronic media has permeated every aspect of our lives [6]. Many prefer to use smartphones or tablets before sleep, which can negatively affect sleep in many aspects, including delayed sleep onset, disrupted sleep patterns, shortened sleep duration, and poor sleep quality [7-10]. Furthermore, problematic use occurs when the behavior surpasses a certain limit. In this study, problematic use of electronic media is not solely determined by the amount of time spent on these platforms, but rather by behavioral indicators that suggest an unhealthy or harmful relationship with them.

Smartphones or tablet use can affect sleep quality in many ways. At first, the use of these devices may directly displace, delay, or interrupt sleep time, resulting in inadequate sleep quantity [11]. The sound of notifications and vibrations of these devices may interrupt sleep. Second, the screens of smartphones and tablets emit blue light, which can suppress the production of melatonin, the hormone responsible for regulating sleep-wake cycles [12]. Third, consuming emotionally charged content, such as news, suspenseful movies, or engaging in online arguments, can increase emotional arousal, making it harder to relax and fall asleep. This emotional arousal can also lead to disrupted sleep and nightmares [13]. Finally, the use of electronic devices before bedtime can lead to a delay in bedtime and a shortened sleep duration, as individuals may lose track of time while engaging with their devices. This can result in a disrupted sleep routine and decreased sleep quality [14].

Some studies have conducted meta-analyses on screen media use and sleep outcomes in 2016, 2019, and 2021 [15-17]. However, these studies had their own limitations. First, the sample size included in their meta-analyses was small (around 10). Second, these studies only focused on 1 aspect of the effect of digital media on sleep quality. For example, Carter et al [16] focused only on adolescents, and both Alimoradi et al [15] and Kristensen et al [17] only reviewed the relationship between problematic use of digital media or devices and sleep quality. Despite of the high heterogeneity found in the meta-analyses, none have compared the effects of different digital media or devices. This study aims to clarify and compare the effects of these different channels.

## Methods

### Literature Search

The research adhered to Preferred Reporting Items for Systematic Reviews and Meta-Analyses (PRISMA) guidelines (Multimedia Appendix 1) and followed a predetermined protocol [18,19]. As the idea and scope of this study evolved over time, the meta-analysis was not preregistered. However, the methodology was defined a priori and strictly followed to reduce biases, and the possible influence of post hoc decisions was minimized. All relevant studies in English, published from January 1, 2018, to October 9, 2023, were searched. We searched the following databases: Web of Science, MEDLINE, PsycINFO, PubMed, Science Direct, Scopus, and Google Scholar. The abstracts were examined manually. The keywords used to search were the combination of the following words: “sleep” OR “sleep duration” OR “sleep quality” OR “sleep problems” AND “electronic media” OR “smartphone” OR “tablet” OR “social media” OR “Facebook” OR “Twitter” OR “online gaming” OR “internet” OR “addiction” OR “problematic” (Multimedia Appendix 2). Additionally, the reference lists of relevant studies were examined.

Two trained coders independently screened the titles and abstracts of the identified papers for eligibility, followed by a full-text review of the selected studies. Discrepancies between the coders were resolved through discussion until a consensus was reached. The reference lists of the included studies were also manually screened to identify any additional relevant studies. Through this rigorous process, we ensured a comprehensive and replicable literature search that could contribute to the robustness of our meta-analysis findings.

### Inclusion or Exclusion Criteria

Titles and abstracts from search results were scrutinized for relevance, with duplicates removed. Full texts of pertinent papers were obtained, and their eligibility for inclusion was evaluated. We mainly included correlational studies that used both continuous measures of time spent using electronic media use and sleep quality. Studies must have been available in English. Four criteria were used to screen studies: (1) only peer-reviewed empirical studies, published in English, were considered for inclusion in the meta-analysis; (2) the studies should report quantitative statistics on electronic media use and sleep quality, including sample size and essential information to calculate the effect size, and review papers, qualitative studies, case studies, and conference abstracts were excluded; (3) studies on both general use and problematic use of electronic media or devices should be included; and (4) only studies that used correlation, regression, or odds ratio were included to ensure consistency.

### Study Coding

Two trained coders were used to code the characteristics of the studies independently. Discrepancies were discussed with the first author of the paper to resolve. Sample size and characteristics of participants were coded: country, female ratio, average age, publication year, and electronic types. Effect sizes were either extracted directly from the original publications or manually calculated. If a study reported multiple dependent effects, the effects were merged into one. If a study reported multiple independent effects from different samples, the effects were included separately. Additionally, to evaluate the study quality, the papers were classified into 3 tiers (high, middle, and low) according to *Journal Citation Reports 2022*, a ranking of journals based on their impact factor as reported in the Web of Science. The few unindexed papers were rated based on their citation counts as reported in Google Scholar.

### Meta-Analysis and Moderator Analyses

The effect sizes were calculated using the correlation coefficient (*r*) as a standardized measure of the relationship between electronic media or device use and sleep quality across studies. When studies reported multiple effect sizes, we selected the one that best represented the overall association between electronic media use and sleep quality. If studies did not provide correlation coefficients, we converted other reported statistics (eg, standardized regression coefficients) into correlation coefficients using established formulas. Once calculated, the correlation coefficients were transformed into Fisher *z* scores to stabilize the variance and normalize the distribution.

Previous meta-studies have shown high levels of heterogeneity. Hence, the random effects model was adopted for all analyses. To explore potential factors contributing to the heterogeneity and to further understand the relationship between electronic media use and sleep quality, we conducted moderator analyses. The following categorical and continuous moderators were examined: media types (online gaming, social media, smartphone, or intent), participants’ average age, culture, female ratio, and sleep quality assessment method. For categorical moderators, subgroup analyses were performed, while for continuous moderators, meta-regression analyses were conducted. All analyses were completed in the Comprehensive Meta-Analysis software (version 3.0; Biostat, Inc).

### Publication Bias

We used statistical methods such as funnel plots to assess the presence of asymmetry and a *p*-curve test to test the *p*-hacking problem, which may indicate publication bias. In case of detected asymmetry, we applied techniques such as the trim-and-fill method to adjust the effect size estimates.

By addressing publication bias, we aimed to provide a more accurate and reliable synthesis of the available evidence, enhancing the validity and generalizability of our meta-analytic findings. Nevertheless, it is essential for readers to interpret the results cautiously, considering the potential limitations imposed by publication bias and other methodological concerns.

## Results

### Search Findings

**Figure 1:**
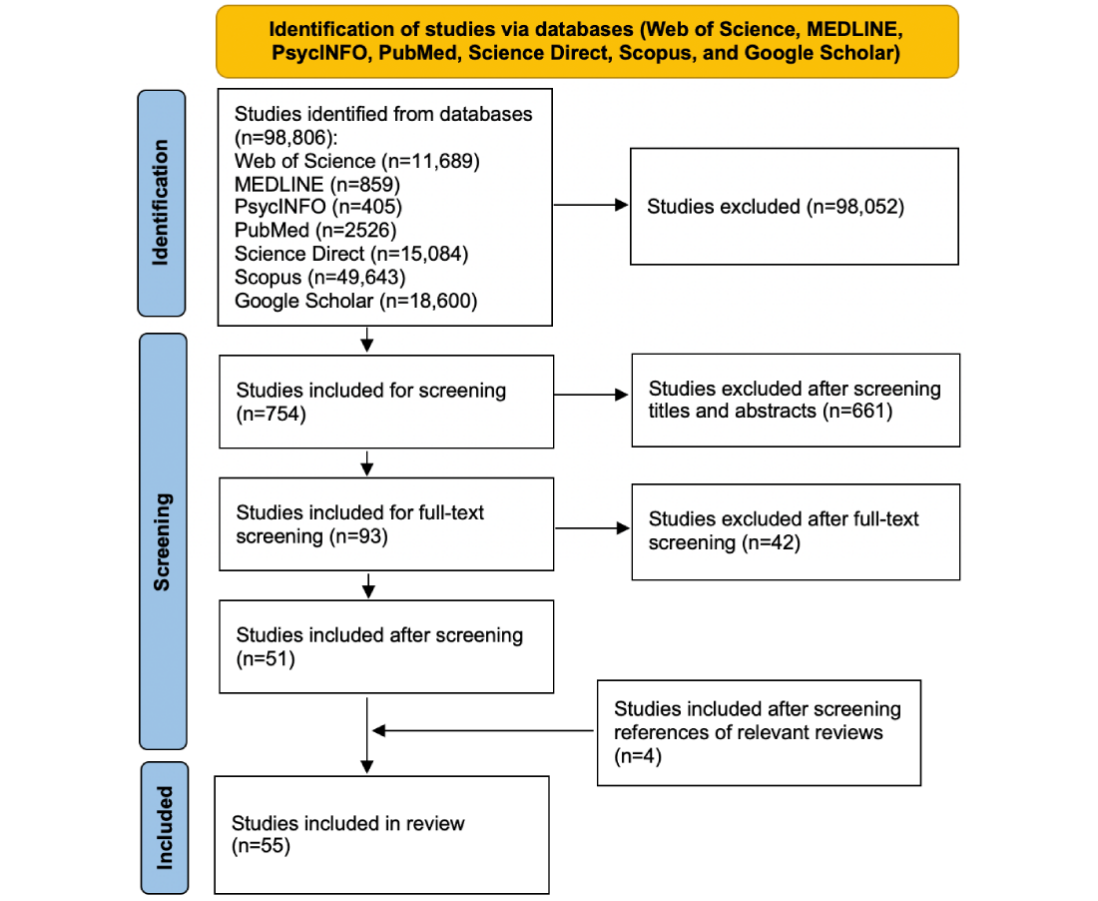
Preferred Reporting Items for Systematic Reviews and Meta-Analyses (PRISMA) flow diagram.

A total of 98,806 studies were identified from databases, especially Scopus (n=49,643), Google Scholar (n=18,600), Science Direct (n=15,084), and Web of Science (n=11,689). Upon removing duplicate records and excluding studies that did not meet the inclusion criteria, 754 studies remained for the screening phase. After screening titles, abstracts, and full texts, 703 studies were excluded. A total of 4 additional studies were identified from the references of relevant reviews. Finally, 55 studies [20-74] were included in the meta-analysis. The flow diagram of the selection is shown in Figure 1.

### Characteristics of Included Studies

In 20 studies, 21,594 participants were included in the analysis of the general use of electronic media and sleep quality. The average age of the sample ranged from 9.9 to 44 years. The category of general online gaming and sleep quality included 4 studies, with 14,837 participants; the category of general smartphone use and sleep quality included 10 studies, with 5011 participants; and the category of general social media use and sleep quality included 6 studies, with 1746 participants.

These studies came from the following countries or areas: Germany, Serbia, Indonesia, India, China, Italy, Saudi Arabia, New Zealand, the United Kingdom, the United States, Spain, Qatar, Egypt, Argentina, and Portugal. The most frequently used measure of electronic media use was the time spent on it. The most frequently used measure of sleep was the Pittsburgh Sleep Quality Index.

In 35 studies, 20,122 participants were included in the analysis of the problematic use of electronic media and sleep quality. The average age of the sample ranged from 14.76 to 65.62 years. The category of problematic online gaming and sleep quality included 5 studies, with 1874 participants; the category of problematic internet use and sleep quality included 2 studies, with 774 participants; the category of problematic smartphone use and sleep quality included 18 studies, with 12,204 participants; and the category of problematic social media use and sleep quality included 11 studies, with 5270 participants. There was a study that focused on both social media and online gaming, which led to its inclusion in the analysis. These studies came from 14 countries or areas: Turkey, the United States, Indonesia, China, France, Taiwan, India, South Korea, Hong Kong, Iran, Poland, Israel, Hungary, and Saudi Arabia. The most frequently used measures of problematic electronic media use were the Internet Gaming Disorder Scale-Short Form, Smartphone Addiction Scale-Short Form, and Bergen Social Media Addiction Scale.

With respect to study quality, the 56 papers were published in 50 journals, 41 of which were indexed in *Journal Citation Reports 2022*, while the remaining 9 journals were rated based on their citation counts as reported in Google Scholar. As a result, of the 56 papers included in the study, 22 papers were assigned a high rating, 18 papers were assigned a middle rating, and 16 papers were assigned a low rating. More information about the included studies is listed in Multimedia Appendix 3 [20-74].

### Meta-Analysis

The results of the meta-analysis of the relationship between general electronic media use and sleep quality showed that electronic media use was associated with a significant decrease in sleep quality (*P*<.001). The pooled effect size was 0.28 (95% CI 0.21-0.35; *k*=20), indicating that individuals who used electronic media more frequently were generally associated with more sleeping problems.

The second meta-analysis showed that problematic electronic media use was associated with a significant increase in sleep problems (*P*≤.001). The pooled effect size was 0.33 (95% CI 0.28-0.38; *k*=36), indicating that participants who used electronic media more frequently were more likely to have more sleep problems.

### Moderator Analyses

At first, we conducted subgroup analyses for different media or devices. The results are shown in Tables 1 and 2. The effect of the relationship between general online gaming and sleep problems was *r*=0.14 (95% CI 0.06-0.22); the effect of the relationship between general smartphone use and sleep problems was *r*=0.33 (95% CI 0.27-0.40); and the effect of the relationship between general social media use and sleep problems was *r*=0.28 (95% CI 0.21-0.34). There are significant differences among these groups (*Q*_between_=14.46; *P*=.001).

**Table 1:** The association of general use of electronic media use and sleep problems.

Media or device types	*k*	*r* (95% CI)	*P* value	*I* ^2^
Online gaming	4	0.142 (0.063-0.219)	<.001	90.953
Smartphone	10	0.334 (0.270-0.396)	<.001	82.437
Social media	6	0.277 (0.209-0.342)	<.001	47.067

**Table 2:** The association of problematic use of electronic media use and sleep problems.

Media or device types	*k*	*r* (95% CI)	*P* value	*I* ^2^
Online game	5	0.494 (0.227-0.691)	.001	97.491
Internet	2	0.514 (0.430-0.589)	<.001	38.784
Smartphone	18	0.250 (0.195-0.303)	<.001	88.754
Social media	11	0.347 (0.288-0.403)	<.001	80.835

The effect of the relationship between problematic gaming and sleep problems was *r*=0.49, 95% CI 0.23-0.69; the effect of the relationship between problematic internet use and sleep problems was *r*=0.51 (95% CI 0.43-0.59); the effect of the relationship between problematic smartphone use and sleep problems was *r*=0.25 (95% CI 0.20-0.30); and the effect of the relationship between problematic social media use and sleep problems was *r*=0.35 (95% CI 0.29-0.40). There are significant differences among these groups (*Q*_between_=27.37; *P*<.001).

We also used age, gender, and culture as moderators to conduct meta-regression analyses. The results are shown in Tables 3 and 4. Only cultural difference in the relationship between Eastern and Western culture was significant (*Q*_between_=6.694; *P*=.01). All other analyses were not significant.

**Table 3:** Culture analysis of general use of electronic media use and sleep problems.

Smartphone			*k*		*r* (95% CI)		*P* value
	Eastern culture	1		0.211 (0.120-0.299)		—^a^	
	Western culture	5		0.322 (0.240-0.399)		.07	

^a^Not applicable.

**Table 4:** Culture analysis of problematic use of electronic media use and sleep problems.

Media or device types		*k*	*r* (95% CI)	*P* value
**Online game**				
	Eastern culture	1	0.249 (0.140-0.352)	—^a^
	Western culture	3	0.461 (0.062-0.733)	.29
**Smartphone**				
	Eastern culture	10	0.231 (0.164-0.295)	—
	Western culture	6	0.287 (0.136-0.425)	.49
**Social media**				
	Eastern culture	5	0.404 (0.333-0.470)	—
	Western culture	4	0.276 (0.208-0.342)	.01

^a^Not applicable.

### Publication Bias

**Figure 2:**
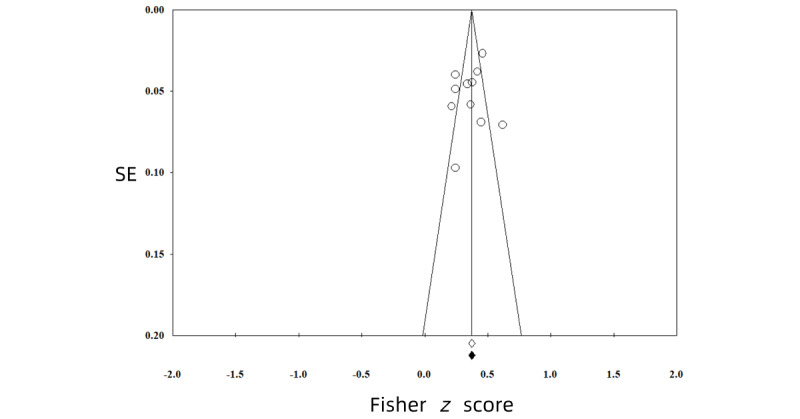
Publication bias of the effect between problematic social media use and sleep problems.

**Figure 3:**
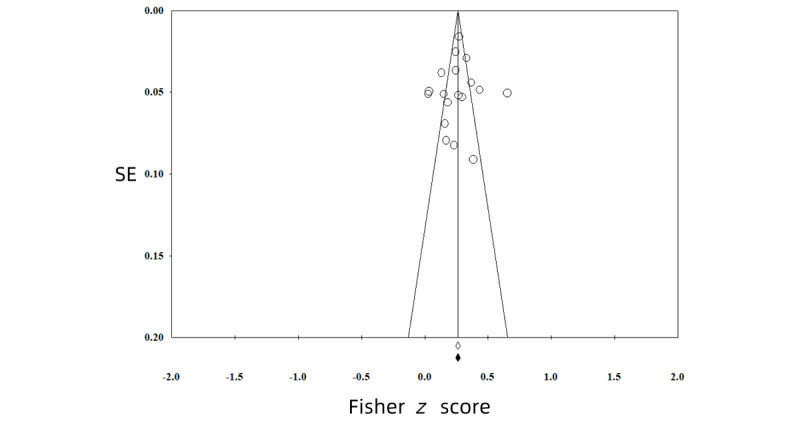
Publication bias of the effect between problematic smartphone use and sleep problems.

**Figure 4:**
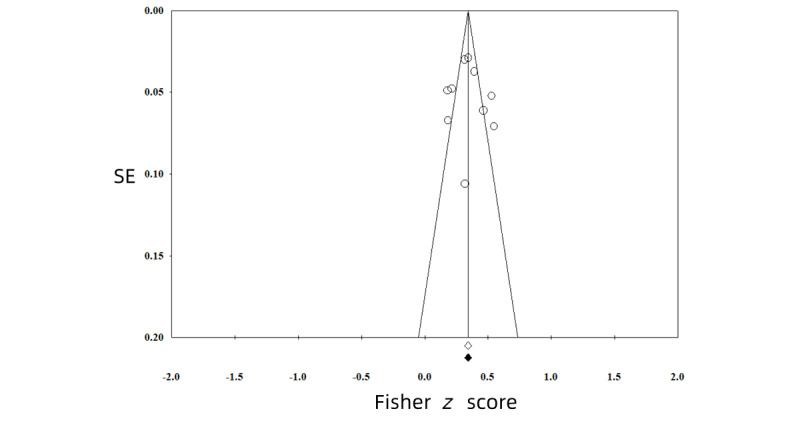
Publication bias of the effect between general smartphone use and sleep problems.

**Figure 5:**
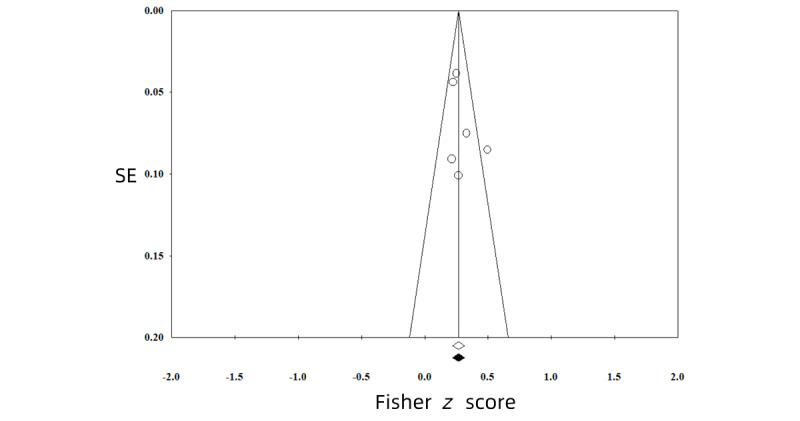
Publication bias of the effect between general social media use and sleep problems.

All funnel plots of the analyses were symmetrical, showing no evidence of publication bias (Figures 2-5). We also conducted *p*-curve analyses to see whether there were any selection biases. The results also showed that there were no biases.

## Discussion

### Principal Findings

This study indicated that electronic media use was significantly linked with decreased sleep quality and increased sleep problems with varying effect sizes across subgroups. General use was associated with a significant decrease in sleep quality. Problematic use was associated with a significant increase in sleep problems. A significant cultural difference was also observed by the meta-regression analysis.

First, there is a distinction in the impact on sleep quality between problematic use and general use, with the former exhibiting a higher correlation strength. However, both have a positive correlation, suggesting that the deeper the level of use, the more sleep-related issues are observed. In addressing this research question, the way in which electronic media use is conceptualized and operationalized may have a bearing on the ultimate outcomes. Problematic use is measured through addiction scales, while general use is predominantly assessed by duration of use (time), leading to divergent results stemming from these distinct approaches. The key takeaway is that each measurement possesses unique strengths and weaknesses, and the pathways affecting sleep quality differ. Consequently, the selection of a measurement approach should be tailored to the specific research question at hand. The duration of general use reflects an individual’s comprehensive involvement with electronic media, and its impact on sleep quality is evident in factors such as an extended time to fall asleep and reduced sleep duration. The addiction scale for problematic use illuminates an individual’s preferences, dependencies, and other associations with electronic media. Its impact on sleep quality is evident through physiological and psychological responses, including anxiety, stress, and emotional reactions.

Second, notable variations exist in how different types of electronic media affect sleep quality. In general, the positive predictive effects of smartphone, social media, and online gaming use durations on sleep problems gradually decrease. In the problematic context, the intensity of addiction to the internet and online gaming has the most significant positive impact on sleep problems, followed by social media, while smartphones exert the least influence. On one hand, longitudinal comparisons within the same context reveal that the content and format of electronic media can have varying degrees of negative impact on sleep quality, irrespective of whether it involves general or problematic use. On the other hand, cross-context comparisons suggest that both general and problematic use play a role in moderating the impact of electronic media types on sleep quality. As an illustration, problematic use reinforces the positive impact of online gaming and social media on sleep problems, while mitigating the influence of smartphones. Considering smartphones as electronic media, an extended duration of general use is associated with lower sleep quality. However, during problematic use, smartphones serve as the platform for other electronic media such as games and social media, resulting in a weakened predictive effect on sleep quality. Put differently, in the context of problematic use, the specific type of electronic media an individual consumes on their smartphones becomes increasingly pivotal in shaping sleep quality.

Third, cultural differences were found to be significant moderators of the relationship between electronic media use and sleep problems in both our study and Carter et al [16]. Kristensen et al [17], however, did not specifically address the role of cultural differences but revealed that there was a strong and consistent association between bedtime media device use and sleep outcomes across the studies included. Our findings showed that the association between problematic social media use was significantly larger in Eastern culture. We speculate that the difference may be attributed to cultural differences in social media use patterns, perceptions of social norms and expectations, variations in bedtime routines and habits, and diverse coping mechanisms for stress. These speculations warrant further investigation to understand better the underlying factors contributing to the observed cultural differences in the relationship between social media use and sleep quality.

Fourth, it was observed that gender and age had no significant impact on sleep quality. The negative effects of electronic media use are not only confined to the sleep quality of adults, and the association with gender differences remains unclear. Recent studies point out that electronic media use among preschoolers may result in a “time-shifting” process, disrupting their sleep patterns [75]. Similarly, children and adolescent sleep patterns have been reported to be adversely affected by electronic media use [76-78]. These findings underscore the necessity of considering age group variations in future research, as electronic media use may differently impact sleep quality across age demographics.

In conclusion, our study, Carter et al [16], and Kristensen et al [17] collectively emphasize the importance of understanding and addressing the negative impact of electronic media use, particularly problematic online gaming and smartphone use, on sleep quality and related issues. Further research is warranted to explore the underlying mechanisms and specific factors contributing to the relationship between electronic media use and sleep problems.

### Strengths and Limitations

Our study, supplemented with research by Carter et al [16] and Kristensen et al [17], contributes to the growing evidence supporting a connection between electronic media use and sleep quality. We found that both general and problematic use of electronic media correlates with sleep issues, with the strength of the correlation varying based on the type of electronic media and cultural factors, with no significant relationship observed with age or gender.

Despite the vast amount of research on the relationship between electronic media use and sleep, several gaps and limitations still exist.

First, the inclusion criteria were restricted to English-language, peer-reviewed empirical studies published between January 2018 and October 2023. This may have led to the exclusion of relevant studies published in other languages or before 2018, potentially limiting the generalizability of our findings. Furthermore, the exclusion of non–peer-reviewed studies and conference abstracts may have introduced publication bias, as significant results are more likely to be published in peer-reviewed journals.

Second, although we used a comprehensive search strategy, the possibility remains that some relevant studies may have been missed. Additionally, the search strategies were not linked with Medical Subject Headings headers and may not have captured all possible electronic media types, resulting in an incomplete representation of the effects of electronic media use on sleep quality.

Third, the studies included in our meta-analysis exhibited considerable heterogeneity in sample characteristics, electronic media types, and measures of sleep quality. This heterogeneity might have contributed to the variability in effect sizes observed across studies. Although we conducted moderator analyses to explore potential sources of heterogeneity, other unexamined factors may still have influenced the relationship between electronic media use and sleep quality.

Fourth, our meta-analysis relied on the correlation coefficient (*r*) as the primary effect size measure, which may not fully capture the complex relationships between electronic media use and sleep quality. Moreover, the conversion of other reported statistics into correlation coefficients could introduce additional sources of error. The correlational nature of the included studies limited our ability to draw causal inferences between electronic media use and sleep quality. Experimental and longitudinal research designs would provide stronger evidence for the directionality of this relationship.

Given these limitations, future research should aim to include a more diverse range of studies, examine additional potential moderators, and use more robust research designs to better understand the complex relationship between electronic media use and sleep quality.

### Conclusions

In conclusion, our updated meta-analysis affirms the consistent negative impact of electronic media use on sleep outcomes, with problematic online gaming and smartphone use being particularly impactful. Notably, the negative effect of problematic social media use on sleep quality appears more pronounced in Eastern cultures. This research emphasizes the need for public health initiatives to increase awareness of these impacts, particularly for adolescents. Further research, including experimental and longitudinal studies, is necessary to delve deeper into the complex relationship between electronic media use and sleep quality, considering potential moderators like cultural differences.

## Appendix

Multimedia Appendix 1 PRISMA (Preferred Reporting Items for Systematic Reviews and Meta-Analyses) 2020 checklist.

Multimedia Appendix 2 Search strategies.

Multimedia Appendix 3 Characteristics of included studies.

Multimedia Appendix 4 Large language model statement.

## Abbreviations

PRISMA: Preferred Reporting Items for Systematic Reviews and Meta-Analyses

## Data Availability

The data sets analyzed during this study are available from the corresponding author on reasonable request.

## References

[1] Brink-Kjaer A, Leary EB, Sun H, Westover MB, Stone KL, Peppard PE, Lane NE, Cawthon PM, Redline S, Jennum P, Sorensen HBD, Mignot E (2022). Age estimation from sleep studies using deep learning predicts life expectancy. NPJ Digit Med.

[2] Killgore WDS (2010). Effects of sleep deprivation on cognition. Prog Brain Res.

[3] Lee S, Mu CX, Wallace ML, Andel R, Almeida DM, Buxton OM, Patel SR (2022). Sleep health composites are associated with the risk of heart disease across sex and race. Sci Rep.

[4] Prather AA, Grandner MA (2019). Sleep, stress, and immunity. Sleep and Health, 1st Edition.

[5] Scott AJ, Webb TL, Martyn-St James M, Rowse G, Weich S (2021). Improving sleep quality leads to better mental health: a meta-analysis of randomised controlled trials. Sleep Med Rev.

[6] Guttmann A (2023). Statista.

[7] Hysing M, Pallesen S, Stormark KM, Jakobsen R, Lundervold AJ, Sivertsen B (2015). Sleep and use of electronic devices in adolescence: results from a large population-based study. BMJ Open.

[8] Lavender RM (2015). Electronic media use and sleep quality. Undergrad J Psychol.

[9] Exelmans L, Van den Bulck J (2016). Bedtime mobile phone use and sleep in adults. Soc Sci Med.

[10] Twenge JM, Krizan Z, Hisler G (2017). Decreases in self-reported sleep duration among U.S. adolescents 2009-2015 and association with new media screen time. Sleep Med.

[11] Exelmans L (2019). Electronic media use and sleep: a self-control perspective. Curr Sleep Med Rep.

[12] Jniene A, Errguig L, El Hangouche AJ, Rkain H, Aboudrar S, El Ftouh M, Dakka T (2019). Perception of sleep disturbances due to bedtime use of blue light-emitting devices and its impact on habits and sleep quality among young medical students. Biomed Res Int.

[13] Munezawa T, Kaneita Y, Osaki Y, Kanda H, Minowa M, Suzuki K, Higuchi S, Mori J, Yamamoto R, Ohida T (2011). The association between use of mobile phones after lights out and sleep disturbances among Japanese adolescents: a nationwide cross-sectional survey. Sleep.

[14] Smith LJ, Gradisar M, King DL, Short M (2017). Intrinsic and extrinsic predictors of video-gaming behaviour and adolescent bedtimes: the relationship between flow states, self-perceived risk-taking, device accessibility, parental regulation of media and bedtime. Sleep Med.

[15] Alimoradi Z, Lin CY, Broström A, Bülow PH, Bajalan Z, Griffiths MD, Ohayon MM, Pakpour AH (2019). Internet addiction and sleep problems: a systematic review and meta-analysis. Sleep Med Rev.

[16] Carter B, Rees P, Hale L, Bhattacharjee D, Paradkar MS (2016). Association between portable screen-based media device access or use and sleep outcomes: a systematic review and meta-analysis. JAMA Pediatr.

[17] Kristensen JH, Pallesen S, King DL, Hysing M, Erevik EK (2021). Problematic gaming and sleep: a systematic review and meta-analysis. Front Psychiatry.

[18] Moher D, Liberati A, Tetzlaff J, Altman DG, PRISMA Group (2009). Preferred reporting items for systematic reviews and meta-analyses: the PRISMA statement. PLoS Med.

[19] Page MJ, McKenzie JE, Bossuyt PM, Boutron I, Hoffmann TC, Mulrow CD, Shamseer L, Tetzlaff JM, Akl EA, Brennan SE, Chou R, Glanville J, Grimshaw JM, Hróbjartsson A, Lalu MM, Li T, Loder EW, Mayo-Wilson E, McDonald S, McGuinness LA, Stewart LA, Thomas J, Tricco AC, Welch VA, Whiting P, Moher D (2021). The PRISMA 2020 statement: an updated guideline for reporting systematic reviews. BMJ.

[20] Akçay D, Akçay BD (2020). The effect of computer game playing habits of university students on their sleep states. Perspect Psychiatr Care.

[21] Alahdal WM, Alsaedi AA, Garrni AS, Alharbi FS (2023). The impact of smartphone addiction on sleep quality among high school students in Makkah, Saudi Arabia. Cureus.

[22] Alam A, Alshakhsi S, Al-Thani D, Ali R (2023). The role of objectively recorded smartphone usage and personality traits in sleep quality. PeerJ Comput Sci.

[23] Almeida F, Marques DR, Gomes AA (2023). A preliminary study on the association between social media at night and sleep quality: the relevance of FOMO, cognitive pre-sleep arousal, and maladaptive cognitive emotion regulation. Scand J Psychol.

[24] Alshobaili FA, AlYousefi NA (2019). The effect of smartphone usage at bedtime on sleep quality among Saudi non-medical staff at King Saud University Medical City. J Family Med Prim Care.

[25] Alsulami A, Bakhsh D, Baik M, Merdad M, Aboalfaraj N (2019). Assessment of sleep quality and its relationship to social media use among medical students. Med Sci Educ.

[26] Altintas E, Karaca Y, Hullaert T, Tassi P (2019). Sleep quality and video game playing: effect of intensity of video game playing and mental health. Psychiatry Res.

[27] Asbee J, Slavish D, Taylor DJ, Dietch JR (2023). Using a frequentist and Bayesian approach to examine video game usage, substance use, and sleep among college students. J Sleep Res.

[28] Bae ES, Kang HS, Lee HN (2020). The mediating effect of sleep quality in the relationship between academic stress and social network service addiction tendency among adolescents. J Korean Acad Community Health Nurs.

[29] Chatterjee S, Kar SK (2021). Smartphone addiction and quality of sleep among Indian medical students. Psychiatry.

[30] Chung JE, Choi SA, Kim KT, Yee J, Kim JH, Seong JW, Seong JM, Kim JY, Lee KE, Gwak HS (2018). Smartphone addiction risk and daytime sleepiness in Korean adolescents. J Paediatr Child Health.

[31] Demir YP, Sumer MM (2019). Effects of smartphone overuse on headache, sleep and quality of life in migraine patients. Neurosciences (Riyadh).

[32] Dewi RK, Efendi F, Has EMM, Gunawan J (2018). Adolescents' smartphone use at night, sleep disturbance and depressive symptoms. Int J Adolesc Med Health.

[33] Eden A, Ellithorpe ME, Meshi D, Ulusoy E, Grady SM (2021). All night long: problematic media use is differentially associated with sleep quality and depression by medium. Commun Res Rep.

[34] Ellithorpe ME, Meshi D, Tham SM (2023). Problematic video gaming is associated with poor sleep quality, diet quality, and personal hygiene. Psychol Pop Media.

[35] Elsheikh AA, Elsharkawy SA, Ahmed DS (2023). Impact of smartphone use at bedtime on sleep quality and academic activities among medical students at Al -Azhar University at Cairo. J Public Health (Berl.).

[36] Gaya AR, Brum R, Brites K, Gaya A, de Borba Schneiders L, Duarte Junior MA, López-Gil JF (2023). Electronic device and social network use and sleep outcomes among adolescents: the EHDLA study. BMC Public Health.

[37] Gezgin DM (2018). Understanding patterns for smartphone addiction: age, sleep duration, social network use and fear of missing out. Cypriot J Educ Sci.

[38] Graham S, Mason A, Riordan B, Winter T, Scarf D (2021). Taking a break from social media improves wellbeing through sleep quality. Cyberpsychol Behav Soc Netw.

[39] Guerrero MD, Barnes JD, Chaput JP, Tremblay MS (2019). Screen time and problem behaviors in children: exploring the mediating role of sleep duration. Int J Behav Nutr Phys Act.

[40] Hamvai C, Kiss H, Vörös H, Fitzpatrick KM, Vargha A, Pikó BF (2023). Association between impulsivity and cognitive capacity decrease is mediated by smartphone addiction, academic procrastination, bedtime procrastination, sleep insufficiency and daytime fatigue among medical students: a path analysis. BMC Med Educ.

[41] Herlache AD, Lang KM, Krizan Z (2018). Withdrawn and wired: problematic internet use accounts for the link of neurotic withdrawal to sleep disturbances. Sleep Sci.

[42] Huang Q, Li Y, Huang S, Qi J, Shao T, Chen X, Liao Z, Lin S, Zhang X, Cai Y, Chen H (2020). Smartphone use and sleep quality in chinese college students: a preliminary study. Front Psychiatry.

[43] Hussain Z, Griffiths MD (2021). The associations between problematic social networking site use and sleep quality, attention-deficit hyperactivity disorder, depression, anxiety and stress. Int J Ment Health Addict.

[44] Imani V, Ahorsu DK, Taghizadeh N, Parsapour Z, Nejati B, Chen HP, Pakpour AH (2022). The mediating roles of anxiety, depression, sleepiness, insomnia, and sleep quality in the association between problematic social media use and quality of life among patients with cancer. Healthcare (Basel).

[45] Jeong CY, Seo YS, Cho EH (2018). The effect of SNS addiction tendency on trait-anxiety and quality of sleep in university students'. J Korean Clin Health Sci.

[46] Karaş H, Küçükparlak İ, Özbek MG, Yılmaz T (2023). Addictive smartphone use in the elderly: relationship with depression, anxiety and sleep quality. Psychogeriatrics.

[47] Kater MJ, Schlarb AA (2020). Smartphone usage in adolescents: motives and link to sleep disturbances, stress and sleep reactivity. Somnologie.

[48] Kharisma AC, Fitryasari R, Rahmawati PD (2020). Online games addiction and the decline in sleep quality of college student gamers in the online game communities in Surabaya, Indonesia. Int J Psychosoc Rehabil.

[49] Kumar VA, Chandrasekaran V, Brahadeeswari H (2019). Prevalence of smartphone addiction and its effects on sleep quality: a cross-sectional study among medical students. Ind Psychiatry J.

[50] Lee Y, Blebea J, Janssen F, Domoff SE (2023). The impact of smartphone and social media use on adolescent sleep quality and mental health during the COVID-19 pandemic. Hum Behav Emerg Technol.

[51] Li L, Griffiths MD, Mei S, Niu Z (2020). Fear of missing out and smartphone addiction mediates the relationship between positive and negative affect and sleep quality among Chinese university students. Front Psychiatry.

[52] Li Y, Mu W, Sun C, Kwok SYCL (2023). Surrounded by smartphones: relationship between peer phubbing, psychological distress, problematic smartphone use, daytime sleepiness, and subjective sleep quality. Appl Res Qual Life.

[53] Luo X, Hu C (2022). Loneliness and sleep disturbance among first-year college students: the sequential mediating effect of attachment anxiety and mobile social media dependence. Psychol Sch.

[54] Luqman A, Masood A, Shahzad F, Shahbaz M, Feng Y (2021). Untangling the adverse effects of late-night usage of smartphone-based SNS among university students. Behav Inf Technol.

[55] Aulia A, Pratiwi A, Makhfudli (2020). Relationship intensity of social media use with quality of sleep, social interaction, and self-esteem in urban adolescents in Surabaya. Sys Rev Pharm.

[56] Ozcan B, Acimis NM (2021). Sleep quality in Pamukkale university students and its relationship with smartphone addiction. Pak J Med Sci.

[57] Peltz JS, Bodenlos JS, Kingery JN, Abar C (2023). Psychological processes linking problematic smartphone use to sleep disturbance in young adults. Sleep Health.

[58] Pérez-Chada D, Bioch SA, Schönfeld D, Gozal D, Perez-Lloret S, Sleep in Adolescents Collaborative Study Group (2023). Screen use, sleep duration, daytime somnolence, and academic failure in school-aged adolescents. PLoS One.

[59] Przepiorka A, Blachnio A (2020). The role of Facebook intrusion, depression, and future time perspective in sleep problems among adolescents. J Res Adolesc.

[60] Rudolf K, Bickmann P, Froböse I, Tholl C, Wechsler K, Grieben C (2020). Demographics and health behavior of video game and eSports players in Germany: the eSports study 2019. Int J Environ Res Public Health.

[61] Sami H, Danielle L, Lihi D, Elena S (2018). The effect of sleep disturbances and internet addiction on suicidal ideation among adolescents in the presence of depressive symptoms. Psychiatry Res.

[62] Scott H, Woods HC (2018). Fear of missing out and sleep: cognitive behavioural factors in adolescents' nighttime social media use. J Adolesc.

[63] Spagnoli P, Balducci C, Fabbri M, Molinaro D, Barbato G (2019). Workaholism, intensive smartphone use, and the sleep-wake cycle: a multiple mediation analysis. Int J Environ Res Public Health.

[64] Stanković M, Nešić M, Čičević S, Shi Z (2021). Association of smartphone use with depression, anxiety, stress, sleep quality, and internet addiction. empirical evidence from a smartphone application. Pers Individ Differ.

[65] Tandon A, Kaur P, Dhir A, Mäntymäki M (2020). Sleepless due to social media? investigating problematic sleep due to social media and social media sleep hygiene. Comput Hum Behav.

[66] Wang PY, Chen KL, Yang SY, Lin PH (2019). Relationship of sleep quality, smartphone dependence, and health-related behaviors in female junior college students. PLoS One.

[67] Wang Q, Zhong Y, Zhao G, Song R, Zeng C (2022). Relationship among content type of smartphone use, technostress, and sleep difficulty: a study of university students in China. Educ Inf Technol.

[68] Wong HY, Mo HY, Potenza MN, Chan MNM, Lau WM, Chui TK, Pakpour AH, Lin CY (2020). Relationships between severity of internet gaming disorder, severity of problematic social media use, sleep quality and psychological distress. Int J Environ Res Public Health.

[69] Xie X, Dong Y, Wang J (2018). Sleep quality as a mediator of problematic smartphone use and clinical health symptoms. J Behav Addict.

[70] Yang SY, Chen KL, Lin PH, Wang PY (2019). Relationships among health-related behaviors, smartphone dependence, and sleep duration in female junior college students. Soc Health Behav.

[71] Yıldırım M, Öztürk A, Solmaz F (2023). Fear of COVID-19 and sleep problems in Turkish young adults: mediating roles of happiness and problematic social networking sites use. Psihologija.

[72] Zhai X, Ye M, Wang C, Gu Q, Huang T, Wang K, Chen Z, Fan X (2020). Associations among physical activity and smartphone use with perceived stress and sleep quality of Chinese college students. Mental Health and Physical Activity.

[73] Zhang MX, Wu AMS (2020). Effects of smartphone addiction on sleep quality among Chinese university students: the mediating role of self-regulation and bedtime procrastination. Addict Behav.

[74] Zhang MX, Zhou H, Yang HM, Wu AMS (2021). The prospective effect of problematic smartphone use and fear of missing out on sleep among Chinese adolescents. Curr Psychol.

[75] Beyens I, Nathanson AI (2019). Electronic media use and sleep among preschoolers: evidence for time-shifted and less consolidated sleep. Health Commun.

[76] Mazurek MO, Engelhardt CR, Hilgard J, Sohl K (2016). Bedtime electronic media use and sleep in children with autism spectrum disorder. J Dev Behav Pediatr.

[77] King DL, Delfabbro PH, Zwaans T, Kaptsis D (2014). Sleep interference effects of pathological electronic media use during adolescence. Int J Ment Health Addict.

[78] Kubiszewski V, Fontaine R, Rusch E, Hazouard E (2013). Association between electronic media use and sleep habits: an eight-day follow-up study. Int J Adolesc Youth.

