# Supplementary material for: Electronic Media Use and Sleep Quality: Updated Systematic Review and Meta-Analysis
Source: J Med Internet Res. 2024 Apr 23;26:e48356. doi: 10.2196/48356 (PMC11077410; doi:10.2196/48356)
Supplement: Multimedia Appendix 2 [file jmir_v26i1e48356_app2.doc]

Multimedia Appendix 2: Search strategies

Web of Science

(((((((((((((((((((((((((((((((((TS=(sleep* electronic media)) OR TS=(sleep duration* electronic media)) OR TS=(sleep quality* electronic media)) OR TS=(sleep problems* electronic media)) OR TS=(sleep* smartphone)) OR TS=(sleep duration* smartphone)) OR TS=(sleep quality* smartphone)) OR TS=(sleep problems* smartphone)) OR TS=(sleep* tablet)) OR TS=(sleep duration* tablet)) OR TS=(sleep quality* tablet)) OR TS=(sleep problems* tablet)) OR TS=(sleep* social media)) OR TS=(sleep duration* social media)) OR TS=(sleep quality* social media)) OR TS=(sleep problems* social media)) OR TS=(sleep* Facebook)) OR TS=(sleep duration* Facebook)) OR TS=(sleep quality* Facebook)) OR TS=(sleep problems* Facebook)) OR TS=(sleep* Twitter)) OR TS=(sleep duration* Twitter)) OR TS=(sleep quality* Twitter)) OR TS=(sleep problems* Twitter)) OR TS=(sleep* online gaming)) OR TS=(sleep duration* online gaming)) OR TS=(sleep quality* online gaming)) OR TS=(sleep problems* online gaming)) OR TS=(sleep* internet)) OR TS=(sleep duration* internet)) OR TS=(sleep quality* internet)) OR TS=(sleep problems* internet)) OR TS=( sleep* addiction* media)) OR TS=( sleep* problematic* media)

Publication Date: 2018-01-01 to 2023-10-09

MEDLINE

SU ( sleep OR sleep duration OR sleep quality OR sleep problems ) AND SU ( electronic media OR smartphone OR tablet OR social media OR Facebook OR Twitter OR online gaming OR internet )

SU ( sleep OR sleep duration OR sleep quality OR sleep problems ) AND SU ( media AND addiction )

SU ( sleep OR sleep duration OR sleep quality OR sleep problems ) AND SU media AND problematic

S1 OR S2 OR S3

Publication Date: 2018-01 to 2023-10

PsycINFO

S1: SU (sleep OR sleep duration OR sleep quality OR sleep problems ) AND SU ( electronic media OR smartphone OR tablet OR social media OR Facebook OR Twitter OR online gaming OR internet)

S2: SU (sleep OR sleep duration OR sleep quality OR sleep problems) AND SU media AND addiction

S3: SU (sleep OR sleep duration OR sleep quality OR sleep problems) AND SU media AND problematic

S1 OR S2 OR S3

Publication Date: 2018-01 to 2023-10

PubMed

2526

(("sleep"[Title/Abstract] OR "sleep duration"[Title/Abstract] OR "sleep quality"[Title/Abstract] OR "sleep problems"[Title/Abstract]) AND ("electronic media"[Title/Abstract] OR "smartphone"[Title/Abstract] OR "tablet"[Title/Abstract] OR "social media"[Title/Abstract] OR "Facebook"[Title/Abstract] OR "Twitter"[Title/Abstract] OR "online gaming"[Title/Abstract] OR "internet"[Title/Abstract])) AND (2018:2023[pdat])

Science Direct

sleep quality AND (electronic media OR smartphone OR tablet OR social media OR Facebook OR Twitter OR online gaming OR internet)

Year: 2018-2023

Article type: Research articles (15,084)

Scopus

( "sleep" OR "sleep duration" OR "sleep quality" OR "sleep problems" ) AND ( "electronic media" OR "smartphone" OR "tablet" OR "social media" OR "Facebook" OR "Twitter" OR "online gaming" OR "internet" ) AND PUBYEAR > 2017 AND PUBYEAR < 2024 AND ( LIMIT-TO ( DOCTYPE , "ar" ) )

Google Scholar

sleep quality and (electronic media OR smartphone OR tablet OR social media OR Facebook OR Twitter OR online gaming OR internet

filter: 2018-2023
