# Supplementary material for: Electronic Media Use and Sleep Quality: Updated Systematic Review and Meta-Analysis
Source: J Med Internet Res. 2024 Apr 23;26:e48356. doi: 10.2196/48356 (PMC11077410; doi:10.2196/48356)
Supplement: Multimedia Appendix 3 [file jmir_v26i1e48356_app3.docx]

**Multimedia Appendix 3. Characteristics of included studies.**

Table S1. Characteristics of included studies (general use)

| Study | Sample size, n | Mean age | Gender (female), n | Type of electronic media | Country/Area | Quality |
| --- | --- | --- | --- | --- | --- | --- |
| Alam et al [22] | 269 | 25.0 | 148 | smartphone | Qatar | Middle |
| Almeida et al [23] | 525 | 22.4 | 442 | social media | Portugal | Low |
| Alshobaili et al [24] | 369 | 27.5 | 155 | smartphone | Saudi Arabia | Low |
| Alsulami et al [25] | 675 | 21.3 | 410 | social media | Saudi Arabia | Low |
| Asbee et al [27] | 1032 | 20.4 | 743 | social media | United States | High |
| Chatterjee et al [29] | 224 | 21.1 | 120 | smartphone | India | Middle |
| Dewi et al [32] | 714 | 16.0 | 410 | smartphone | Indonesia | Middle |
| Elsheikh et al [35] | 1184 | 21.4 | 527 | smartphone | Egypt | High |
| Gaya et al [36] | 1101 | 14.1 | 597 | smartphone | Spain | High |
| Graham et al [38] | 124 | 22.5 | 94 | SNS | New Zealand | High |
| Guerrero et al [39] | 11875 | 9.9 | 5681 | game | United States | High |
| Huang et al [42] | 439 | 18.8 | 381 | smartphone | China | High |
| Kater et al [47] | 201 | 19.2 | 155 | smartphone | Germany | Low |
| Lee et al [50] | 180 | 14.0 | 100 | social media | United States | High |
| Makhfudli et al [55] | 141 | 17.0 | 52 | social media | Indonesia | Low |
| Pérez-Chada et al [58] | 864 | 15.5 | 323 | game | Argentina | Middle |
| Rudolf et al [60] | 1066 | 22.9 | 86 | game | Germany | High |
| Scott et al [62] | 101 | 14.0 | 67 | social media | United Kingdom | Middle |
| Spagnoli et al [63] | 418 | 44.0 | 258 | smartphone | Italy | High |
| Stanković et al [64] | 92 | 21.1 | 74 | smartphone | Serbia | Middle |

Table S2. Characteristics of included studies (problematic use)

| Study | Sample size, n | Mean age | Gender (female), n | Type of electronic media | Country/Area | Quality |
| --- | --- | --- | --- | --- | --- | --- |
| Akçay et al [20] | 892 | 22.8 | 629 | game | Turkey | Middle |
| Alahdal et al [21] | 373 | 15.8 | 127 | smartphone | Saudi Arabia | Low |
| Altintas et al [26] | 217 | 24.4 | 42 | game | French | High |
| Bae et al [28] | 204 | 18.0 | 121 | SNS | South Korea | Low |
| Chung et al [30] | 357 | 15.1 | 233 | smartphone | South Korea | Low |
| Demir et al [31] | 123 | 41.0 | 54 | smartphone | Turkey | Low |
| Eden et al [33] | 109 | 20.8 | 57 | social media | United States | Low |
| Ellithorpe et al [34] | 354 | 21.0 | 144 | game | United States | Middle |
| Gezgin [37] | 161 | 16.2 | 67 | smartphone | Turkey | High |
| Hamvai et al [40] | 211 | 22.2 | 151 | smartphone | Hungary | Middle |
| Herlache et al [41] | 143 | 19.9 | 110 | Internet | United States | Low |
| Hussain et al [43] | 638 | 32.0 | 304 | SNS | United States | High |
| Imani et al [44] | 288 | 52.3 | NR | social media | Iran | Middle |
| Jeong et al [45] | 213 | 25.0 | 96 | SNS | South Korea | Low |
| Karaş et al [46] | 392 | 65.6 | 89 | smartphone | Turkey | Low |
| Kharisma et al [48] | 111 | 22.5 | 43 | game | Indonesia | Middle |
| Kumar et al [49] | 150 | 20.0 | 88 | smartphone | India | High |
| Li et al [51] | 1164 | 20.1 | 656 | smartphone | China | High |
| Li et al [52] | 742 | 15.4 | 395 | smartphone | China | Middle |
| Luo et al [53] | 487 | 18.2 | 205 | social media | China | Low |
| Luqman et al [54] | 701 | 22.0 | 337 | SNS | China | Middle |
| Ozcan et al [56] | 1545 | 21.4 | 878 | smartphone | Turkey | Low |
| Peltz et al [57] | 385 | 20 | 312 | smartphone | United States | Middle |
| Przepiorka et al [59] | 426 | 14.7 | 209 | social media | Poland | Middle |
| Sami et al [61] | 631 | 15.0 | 344 | Internet | Israel | High |
| Tandon et al [65] | 1398 | 23.0 | 802 | social media | India | High |
| Wang et al [66] | 512 | 20.0 | 172 | smartphone | China | High |
| Wang et al [67] | 409 | 17.4 | 409 | smartphone | Taiwan | Middle |
| Wong et al [68] | 300 | 20.9 | 178 | social media | Hong Kong | High |
| Wong et al [68] | 300 | 20.9 | 178 | game | Hong Kong | High |
| Xie et al [69] | 686 | 14.8 | 382 | smartphone | China | High |
| Yang et al [70] | 385 | 17.5 | 385 | smartphone | Taiwan | Middle |
| Yıldırım et al [71] | 506 | 28.2 | 342 | social media | Turkey | Low |
| Zhai et al [72] | 3864 | 19.5 | 1197 | smartphone | China | High |
| Zhang et al [73] | 427 | 19.4 | 282 | smartphone | China | High |
| Zhang et al [74] | 318 | 16.9 | 204 | smartphone | China | Middle |
