# Supplementary material for: Electronic Media Use and Sleep Quality: Updated Systematic Review and Meta-Analysis
Source: J Med Internet Res. 2024 Apr 23;26:e48356. doi: 10.2196/48356 (PMC11077410; doi:10.2196/48356)
Supplement: Multimedia Appendix 4 [file jmir_v26i1e48356_app4.pdf]

Large Language Model Statement:

In the process of writing this paper, we utilized the GPT-4 model as an editing tool. The GPT-4 model assisted us in enhancing the fluency and readability of the text while preserving the original meaning. However, all research content, data analysis, and conclusions were independently completed by the authors. The GPT-4 model did not contribute to the generation of any original content.

One GPT prompt example for proofreading is "Can you improve the following paragraph to make it more academic: 'Our study, supplemented with research by Carter and Kristensen, contributes to the growing evidence supporting a connection between electronic media use and sleep quality. We found that both general and problematic use of electronic media correlates with sleep issues, with the strength of the correlation varying based on the type of electronic media and cultural factors.' Please sharpen the topic sentence, reduce repetition of words and ideas, and maintain the current tone, but improve the text overall to make it sound more academic to the standard of expert writing."

TE **您**

Can you improve the following paragraph to make it more academic: "Our study, supplemented with research by Carter and Kristensen, contributes to the growing evidence supporting a connection between electronic media use and sleep quality. We found that both general and problematic use of electronic media correlates with sleep issues, with the strength of the correlation varying based on the type of electronic media and cultural factors. ". Please sharpen the topic sentence, reduce repetition of words and ideas, and maintain the current tone, but improve the text overall to make it sound more academic to the standard of expert writing.

ChatGPT

This study, augmented by the findings of Carter and Kristensen, adds to the accumulating body of evidence that suggests a link between the use of electronic media and sleep quality. Our analysis reveals that both general and problematic engagement with electronic media is associated with sleep disturbances. Moreover, the magnitude of this correlation appears to vary according to the specific type of electronic media employed and is influenced by cultural factors.

‹ 2 / 2 ›
